# Exploring the motor skill proficiency barrier among children with intellectual disabilities: Analysis at a behavioural component level

**DOI:** 10.1371/journal.pone.0288413

**Published:** 2023-11-28

**Authors:** Hayley Kavanagh, Johann Issartel, Sarah Meegan, Mika Manninen

**Affiliations:** 1 Faculty of Science and Health, School of Health and Human Performance, Dublin City University, Dublin, Ireland; 2 Special Olympics Ireland, Sport Ireland Campus, Dublin, Ireland; 3 MoveAhead Limited, Guinness Enterprise Center, Dublin, Ireland; Università degli Studi di Milano: Universita degli Studi di Milano, ITALY

## Abstract

Models of childhood motor development began to emerge in the 1960’s. Since then, numerous models have proposed the importance of obtaining a proficient level of fundamental movement skill (FMS) competence during childhood and deemed it to be critical for participation in lifelong sports and physical activity. This study examined FMS at the behavioural component level in children with intellectual disabilities (CwID) (n = 100, 60% boys, aged 5–12 years). Participants were assessed using the Test of Gross Motor Development 3^rd^ edition (TGMD-3) and the balance subtest from Bruininks-Oseretsky Test of Motor Proficiency 2 (BOT-2). For the whole sample, 0% participants mastered all 10 FMS, 1% (n = 1) participants mastered all 4 locomotor skills while 0% (n = 100) participants mastered all ball skills. A multiple regression was carried out to investigate whether the interaction of gender and age was a predictor of FMS proficiency. Linear regressions were also carried out to investigate whether gender or age was a predictor of FMS proficiency. The results presented will help to identify weaknesses in skills at the behavioural component level and will enable researchers and practitioners to address low levels of motor skill proficiency among CwID.

## Introduction

Models of childhood motor development began to emerge in the 1960’s. Since then, numerous models (e.g. Seefeldt’s Motor Skill Proficiency Barrier, Clark and Metcalfe’s Mountain of Motor Development and Gallahue’s Hourglass Model of Motor Development) have proposed the importance of obtaining a proficient level of fundamental movement skills (FMS) competence during childhood and deemed it to be critical for participation in lifelong sports and physical activity [1–5]. The development of movement skills described by these models follows a hierarchical structure [1–5] which previous exposure and practice of FMS directly influences performance and further learning or progression [6]. This is demonstrated by the commonly regarded interdependent phases of the motor development pathway beginning at FMS, leading to transitional movement skills (TMS) then onto sports specific skills (SSS) [5,7]. TMS are those which “assist with the transition from basic patterns to context-specific use of skills in games and activities’’ [8] eg. Non sport specific skills (jump rope), small sided games and lead up activities (eg. Putt a ball into a target—golf), while examples of SSS include dribbling in basketball, volleying in tennis and a back-pass in rugby [7].

FMS are believed to be the ‘building blocks’ required for performing activities [9,10]. Children are not born with the ability to move proficiently. It is essential to give them opportunities to practice, learn and reinforce FMS overtime [1,9,11–13]. If children cannot skip, run, kick, throw, catch etc., they will be presented with limited opportunities to participate in physical activity as they get older because they will not possess the prerequisite skills to be active [14,15]. Seefeldt’s model indicated that children with low FMS proficiency will experience difficulties learning TMS [5]. This ‘glass ceiling’ is known as the proficiency barrier.

Children with intellectual disabilities (CwID) are a cohort who consistently demonstrate low FMS proficiency in the literature [16,17], thus we can surmise that this population will be significantly impacted by the proficiency barrier. In order to determine how far below the proficiency barrier CwID are, it is first important to investigate FMS proficiency at both an individual skill and behavioural level basis. In essence, each FMS is composed of multiple behavioural components which are deemed essential to successfully and competently perform the skill (e.g. Ulrich’s Test of Gross Motor Development (TGMD-3)). Behavioural components can also be described as ‘performance criteria’ [18], achieving good proficiency in these performance criteria demonstrates a mature movement skill pattern [19]. Identification of weaknesses in skills at the behavioural component level enable researchers and practitioners to address low levels of motor skill proficiency [20,21].

The majority of studies that document FMS proficiency of CwID, present the data as overall levels of FMS proficiency [17,22–24], with a significant lack of data documenting FMS proficiency at the behavioural component level of performance. Exclusively reporting FMS at this aggregated level has left a knowledge gap in regard to CwID motor development, as many of the behavioural components are interlinked across multiple FMS, which if investigated further could demonstrate a trend of similar FMS deficiency across skills [19]. Hence, researchers, coaches and teachers cannot determine what individual skills or skill characteristics remain underdeveloped [25].

To date, among typically developing children (TDC) only five studies have analysed FMS proficiency at both a skill and behavioural component level [19,25–28]. To the best of our knowledge, no studies have yet been conducted to examine the individual skills and behavioural components of FMS proficiency in CwID. Reporting FMS at both an individual skills and behavioural component level would broaden the knowledge and understanding of FMS proficiency [25]. This would enable researchers to provide coaches and teachers with important information regarding CwID’s development characteristics [25,29]. In addition to supporting gatekeepers to develop appropriate evidence-based strategies to address low FMS proficiency among CwID by focusing on activities that aim to develop and improve weaker FMS at a behavioural component level [25].

The aim of this study is threefold: (1) to to assess FMS at the behavioural component level of performance among CwID, (2) to identify weaknesses within performance and commonality of these weaknesses across skills and (3) to investigate the role of gender and age on FMS proficiency for CwID.

## Method

### Participants

Cross-sectional data were collected as part of the *‘SO Fun’* project with Special Olympics Ireland. Fifteen Special Olympics Young Athletes clubs were contacted with 10 clubs agreeing to participate in the study. The eligibility criteria for participating in this study included; children with intellectual disabilities who are registered with the Special Olympics Young Athletes programme, aged 4–12 years, who are fully mobile and can walk without the use of an aid. A sample of 100 children with intellectual disabilities were recruited from clubs across 8 counties in each of the four provinces of Ireland and Northern Ireland. 66% of the participants had Down Syndrome (DS), while the remaining participants reported their condition as an Intellectual Disability. The sample consisted of 60% boys with an age range of 4–12 years and a mean age of 7.53 ± 2.01. Data were collected during the period of October 2021 to June 2022.

Ethical approval was obtained from Dublin City University, Research Ethics Committee (DCUREC/2021/100). The coaches of each of the participating clubs provided initial written consent for the research team to visit the club, while written parental/guardian consent was also obtained and required in order for participants to partake in the study as they were minors. Written consent forms were collected by the research team prior to data collection. Anonymity was maintained with each participant assigned a unique numerical code.

### Measures

Participants demographics including age and gender were collected through the consent forms and questionnaires completed by parents. The FMS proficiency of the participants was assessed using a subset of the process-oriented battery, the Test of Gross Motor Development-3rd Edition (TGMD-3). The TGMD-3 was individually administered to each participant, the skills focused on two subsets of FMS, locomotor skills (run, skip, horizontal jump and hop) and ball skills (catch, kick, overhand throw, underhand throw, stationary dribble and one hand strike) [18].

Balance was assessed using a subtest of the Bruininks-Oseretsky Test of Motor Proficiency 2 Short Form (BOT-2-SF). Participants completed two tasks within the balance component, a single leg stand on a balance beam with the eyes open and walking forward heel-to-toe on the line. The authors chose to use the TGDM-3 and the BOT-2 as the motor competence assessment tools due to their psychometric properties in assessing CwID, particularly in field settings [30].

### Data collection

All members of the research team undertook formal training in order to ensure an in-depth understanding of the skill assessment batteries, in addition to establishing consistency when visually demonstrating the skills to each participant. The visual demonstration of the skill was in line with Ulrich’s [18] and Bruininks [31] protocols. The skill assessment batteries were individually administered to each participant during their Young Athletes club training session. Participants received no cues or verbal feedback. Participants were provided with an opportunity to perform a practice trial to become accustomed with each skill, followed by two opportunities to perform the skill. All of the participants’ performances were video recorded.

A trained member of the research team observed each trial retrospectively, assessed and scored each skill component. A score of 1 was given if the participant successfully performed the criteria and a 0 was recorded if the participant failed to meet the criteria. Participants’ raw scores per skill were calculated by collating the scores from both trials. Once all skills were assessed, raw subtest scores for locomotor and ball skills were calculated and were then combined to provide a total raw FMS score.

The balance subtests were scored based on their performance outcome. Walking forward heel-to-toe on the line was graded based on the number of steps taken by the participant, while adhering to strict criteria [31]. Participants were then awarded points based on the number of successful steps taken, e.g., six continuous steps in line with criteria, equals a max score of four points. The single leg stand on the balance beam was graded on the amount of time the participant could maintain their balance while adhering to the strict criteria [31]. Participants were then awarded points based on the time they maintained their balance, e.g., maintaining balance for 10 seconds in line with the criteria equals a top score of four points. Second trials were only carried out if the maximum score was not reached in the first trial [31].

### Data analysis

All data was analysed using SPSS version 27 and R. To describe the characteristics of the data, means, standard deviations, and tetrachoric correlations on the variables of interest were computed. The main analyses were undertaken on the total FMS scores, locomotor, ball skills and balance subtest scores. Descriptive statistics and frequencies for locomotor and ball skills and their associated behavioural components were calculated. Cohen’s d was used as the effect size in group mean difference measures and an alpha level of .05 was established for all statistical analysis. Additionally, a binary variable composed of “mastery” and “near mastery” was created for each skill and is reported in Table 2 as “% Mastery”. Using procedures previously described by researchers [10,19,26], ‘mastery’ was defined as performing all skill component criteria correctly on both trials, ‘near mastery’ was described as performing all but one skill component criteria correctly per trial but not twice for the same component, while ‘poor’ was described as any participant who scored below these two categories (i.e. their performance was incorrect on two or more skill criteria on both trials) [21]. The proportion of children who achieved mastery in all of the 10 skills was determined. The proportion of children not achieving mastery in any of the skills was also determined. The number of skills mastered per participant was calculated. The percentage of boys and girls who achieved mastery/ near mastery in each skill was reported by producing descriptive statistics and frequency tables (procedure described below). Tetrachoric correlation coefficients were computed with two binary variables, Prevalence of Failure (Classified as ‘Poor’, ‘Near Mastery’) and Mastery, to determine correlations at an individual level between behavioural components of each skill. These findings are presented in a tetrachoric correlation matrix (Table 2). Finally, a multiple regression was used to determine whether the interaction of age and gender was a predictor of FMS proficiency. Simple linear regressions were used to assess the impact of gender and age on participants’ locomotor, ball skills, balance and total FMS proficiency.

## Results

### Behavioural component analysis

**Fig 1:**
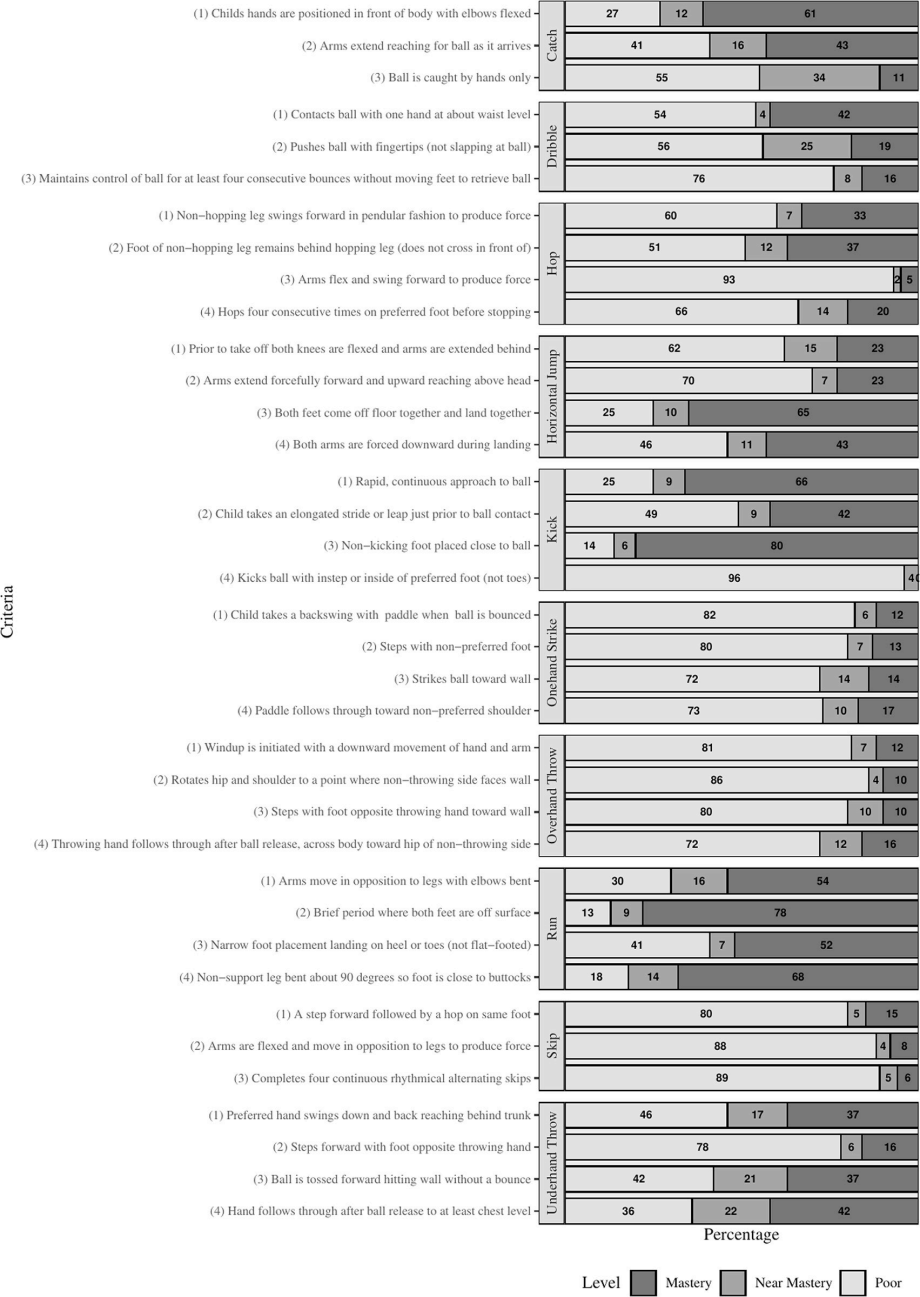
Prevalence of failure (%) (Classified as ‘poor’, ‘near mastery’) and prevalence of mastery (%) for each behavioural component of locomotor and ball skills.

A comprehensive analysis of the behavioural components (i.e. the performance criteria of the movement pattern e.g arms extended, knees flexed etc.) of each individual skill from the locomotor and ball skills subtests were conducted (Figs 1 and S1). For the whole sample, 0% (n = 100) participants mastered all 10 FMS, 1% (n = 1) participants mastered all 4 locomotor skills while 0% (n = 100) participants mastered all ball skills. On an individual skill level, the percentage of participants not achieving mastery in any one skill is 52% (n = 52). On average, each participant mastered 0.91 skills, i.e. less than 1 skill out of 10. A simple tetrachoric correlation matrix of the behavioural components is presented in S1 Fig, highlighting how strong or weak the associations between behavioural components are. A selection of skills with significant positive correlations (≥ .6) with multiple other skill components, were extracted from the larger tetrachoric correlation matrix and are presented in Table 1. A ‘0’ indicates no association while a ‘1’ or ‘-1’ indicates strong positive or negative associations, respectively. The criteria for each skill in the correlation matrix are listed in the same order as outlined in Fig 1.

**Table 1:** Examples of significant tetrachoric correlations between behavioural components of different skills (Skills with significant positive correlations (≥ .6) with multiple other skill components).

	Kick C1	Kick C2	Underhand Throw C1	Underhand Throw C2	Underhand Throw C3
Horizontal Jump C1		0.64			
Horizontal Jump C2	0.60	0.81			
Horizontal Jump C3	0.96	0.70			
Horizontal Jump C4	0.69	0.97			
Run C2	0.60	0.61			0.65
Run C4	0.64	0.61			
Dribble C2			0.62	0.67	
Dribble C3					0.61
One hand Strike C2			0.69	0.62	
One hand Strike C3			0.67	0.67	
Overhand Throw C2				0.60	0.62

C = criteria, p < .01.

### Gender and age

**Fig 2:**
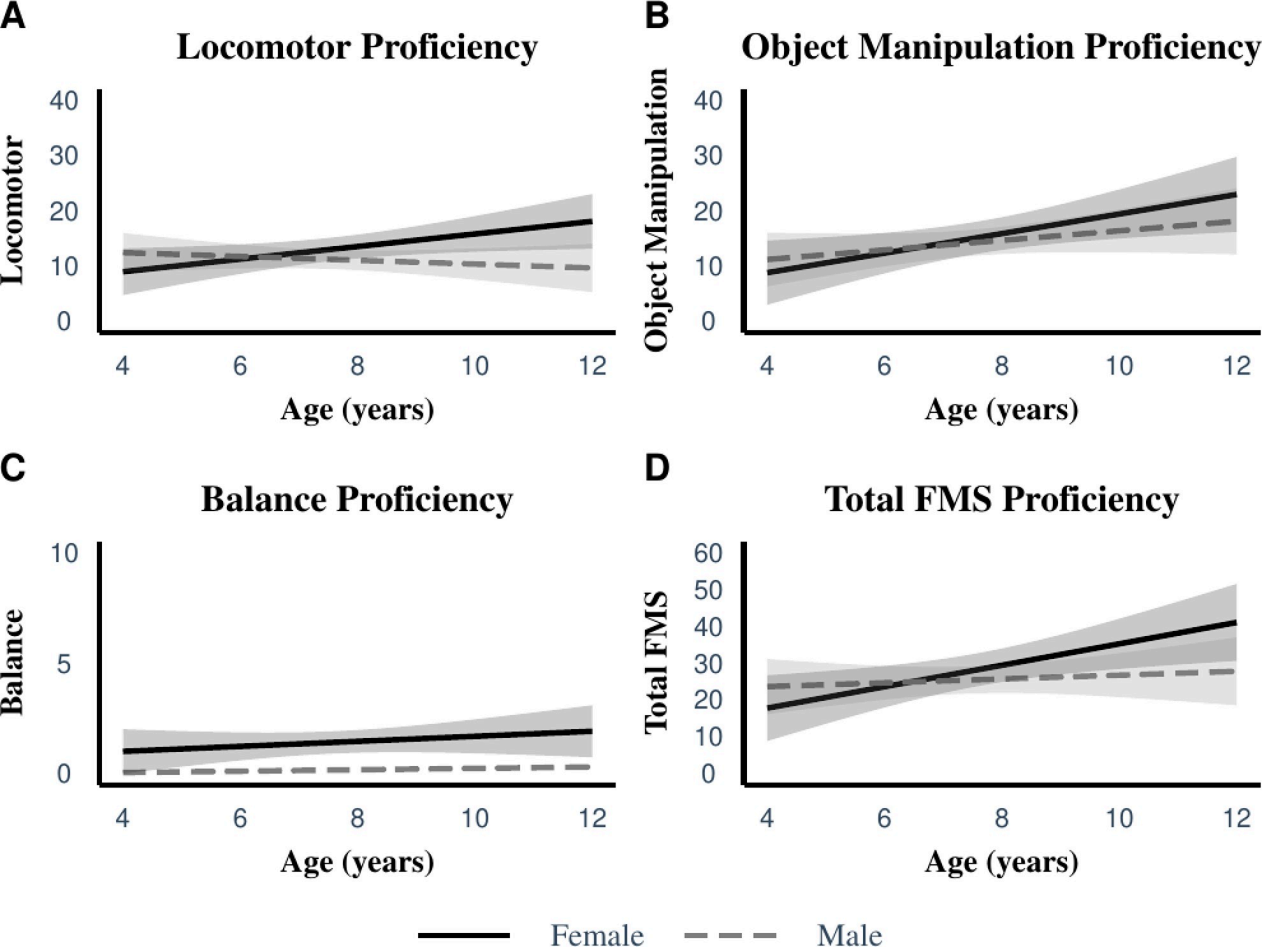
Interaction effect of age and gender on FMS proficiency.

A multiple regression was carried out to investigate whether the interaction of gender and age was a predictor of FMS proficiency. The model demonstrated no significant interaction effect for total FMS proficiency (F(7,83) = 1.1, p = .068, *R*^*2*^ = .15). Similarly, no significant interaction effect was found to predict locomotor F(7,83) = 1.99, p = .067, *R*^*2*^ = .15); ball skills (F(7,83) = 1.5, p = .19, *R*^*2*^ = .12) and balance scores (F(7,83) = .44, p = .88, *R*^*2*^ = .04). Across all of the models, the lack of a significant interaction between age and gender suggests that FMS proficiency remains the same regardless of these variables, i.e. does not change differently between genders as the children age (Fig 2). A linear regression was carried out to investigate if age was a predictor of FMS proficiency. The model demonstrated a significant effect for total FMS proficiency (F(1,98) = 4.8, p = .031, *R*^*2*^ = .047). Similarly, a significant effect was found to predict ball skills (F(1,98) = 7.5, p = .007, *R*^*2*^ = .071). No significant effect was found to predict locomotor (F(1,98) = .81, p = .37, *R*^*2*^ = .008) and balance skills (F(1,98) = .80, p = .38, *R*^*2*^ = .008). Across two of the models, age had a significant effect on total FMS proficiency and ball skills meaning that older children perform better in these two domains compared to younger children.

A linear regression was carried out to investigate if gender was a predictor of FMS proficiency. The model demonstrated no significant effect was found to predict total FMS proficiency (F(1,98) = .9, p = .35, *R*^*2*^ = .009), locomotor (F(1,98) = 1.75, p = .19, *R*^*2*^ = .018) and ball skills (F(1,98) = .25, p = .62, *R*^*2*^ = .003). In contrast to this, a significant effect was found to predict balance skills (F(1,98) = .14.9, p = .000, *R*^*2*^ = .132) with girls outperforming the boys (see Table 2).

**Table 2:** Mean (±SD) FMS proficiency scores and % mastery among CwID.

	Score (M *±SD)*				% Mastery	
FMS	Boys	Girls	Sig.	ES	Boys	Girls
*Locomotor *						
Run	5.37 ± 2.68	5.78 ± 2.14	.42	0.17	56.7	62.5
Skip	0.59 ± 1.35	0.93 ± 1.74	.27	0.22	5.0	12.5
Hop	2 ± 2.38	2.63 ± 2.83	.24	0.25	11.7	22.5
Horizontal Jump	3.32 ± 2.94	3.8 ± 2.33	.39	0.19	28.4	22.5
Total (max score = 30)	11.27 ± 7.16	13.13 ± 6.43	.19	0.28	25.45^#^	30^#^
*Ball Skills*						
Dribble	1.65 ± 2.11	1.78 ± 2.04	.77	0.07	21.7	15.0
Catch	2.94 ± 1.6	3.03 ± 1.77	.79	0.06	38.4	22.5
Kick	4.02 ± 2.24	3.7 ± 2.11	.48	0.15	21.7	22.5
Overhand Throw	1.09 ± 1.91	1.6 ± 2.19	.22	0.25	5.0	7.5
Underhand Throw	3.1 ± 2.66	3.6 ± 2.43	.35	0.2	18.4	25.0
One hand Strike	1.47 ± 2.37	1.53 ± 2.1	.9	0.03	13.4	5.0
Total (max score = 44)	14.25 ± 9.55	15.23 ± 9.62	.62	0.11	19.8^#^	16.25^#^
*Balance*						
Beam Balance	0.14 ± 0.66	0.58 ± 1.18	.02*	0.47	0.00	0.00
Walking on Line	0.04 ± 0.26	0.85 ± 1.48	.001**	0.77		
Total (max score = 8)	0.17 ± 0.7	1.43 ± 2.39	.001**	0.72	0.00^#^	0.00^#^
*FMS Total* (Loco & BS) (max score = 74)	25.52 ± 14.7	28.35 ± 14.6	.35	0.2	22.62^#^	23.12^#^

*p < .05

**p < .01.

ES = Cohen’s d # = Average % Mastery Loco = Locomotor BS = Ball Skills.

Additionally, we examined the degree to which boys and girls with ID “mastered” the individual skills within the locomotor, ball skills and balance subtests. The percentage of mastery/near mastery achieved by the genders (see Table 2). The average percentage mastery for each subtest was calculated by adding the percentage mastery of the individual skills and dividing by the number of skills. The average percentage mastery for total FMS was calculated by adding the average percentage mastery of locomotor and ball skills and dividing by two.

## Discussion

The aim of this study is threefold: (1) to to assess FMS at the behavioural component level of performance among CwID, (2) to identify weaknesses within performance and commonality of these weaknesses across skills and (3) to investigate the role of gender and age on FMS proficiency for CwID.

### Behavioural component analysis

Cumulatively, the findings of the present study suggest that FMS proficiency levels of CwID are only at the initial stage of motor development [2,3,32] with 0% of the participants mastered all ten FMS. Also on average, participants mastered less than one in ten skills, indicating extremely low skill execution among CwID aged 4-12.The results demonstrate that CwID do not have the “building blocks” required to develop and perform more complex FMS, therefore they may potentially be experiencing a proficiency barrier, limiting their ability to progress onto TMS and then SSS. Without achieving adequate proficiency in FMS they limit their ability to participate in lifelong sport and physical activity [14,26,33].

From perspectives rooted in theory, these motor skill delays can be described by Newell’s [34] model of motor development which explains the interaction between an individual’s constraints (e.g. motivation, intellectual functioning, body mass), the task constraints (specific to task being delivered e.g. instructions of task, complexity and goal) and their surrounding environmental constraints (e.g. how others are acting around you, loud noises new environment), all factors which may limit or impair FMS development [25,26,35]. Previous research has found that a constraints-led approach is a practical coaching/teaching method that enables the practitioner to manipulate certain constraints which best allows the learner to develop mature motor skill patterns [34,36]. An example of where this approach has proven successful for CwID (aged 6–10 years) is in a study by Capio and Eugia [37] where they designed a ball skills training program consisting of six skills. The tasks for each skill were adapted to reduce the number of errors experienced and set the child up for success e.g. distance from the target (overhand throw), size of the target (underhand throw), size of the ball, light beach ball—hard rubber (catch), distance from the goal (kick) and number of one hand dribbles before catching ball with two hands (dribble). The results displayed significant and large improvements in ball skills proficiency of CwID [37]. This is an example of the constraints led approach in action where the researchers adapted the task constraint in order to directly impact the motor behaviour.

It can be argued that mastery of the skills demonstrated in this current study were particularly weak due to high failure levels amongst specific behavioural components [19] as demonstrated in Fig 1. While each of the ten FMS demonstrate different outcomes, there is significant overlap between the behavioural components of these skills [19,25,26]. The tetrachoric correlation coefficients presented in Table 1 and S1 Fig provide further insight into the interdependencies of behavioural components across multiple skills and their influence on motor skill development and performance. Examples of these interdependencies include: i) criteria 2 for run and criteria 3 for horizontal jump (S1 Fig) with a strong correlation coefficient of .67; ii) participants who failed to have both of their feet off the ground for a brief period of time in the run, also failed to have both of their feet coming off the floor together and landing together in the horizontal jump and iii) performance in criteria 3 of the horizontal jump is strongly associated with criteria 1 (correlation coefficient .96) and criteria 2 (correlation coefficient .70) in the kick (Table 1). This demonstrates that the behavioural components within the horizontal jump are linked with running and kicking ability. Likewise, criteria 1–3 in the underhand throw (Table 1) has a strong positive performance of criteria 2 and 3 in the overhand throw (correlation coefficient .6) and criteria 2 (correlation coefficient .6) and 3 (correlation coefficient .7) in the one hand strike. This means that if CwID are performing poorly in one or more criteria of a certain skill, this is likely to negatively impact their performance across a number of other skills. Coaches and teachers of CwID should be concerned with the behavioural components which were failed by the majority of participants as this data provides the crucial components of task constraints that interventions must target [25] in order to increase the FMS proficiency of CwID. It is also beneficial for practitioners on the ground to understand the significant overlap between behavioural components of FMS.

Upon further investigation, the behavioural components that are regarded as more difficult to master demonstrated the highest percentage failure. Typically, the performance issues arose when CwID were asked to coordinate movements that involved both sides of their bodies or moving both arms and legs sequentially as part of the overall skill production. Examples of these movements (S1 Fig and Table 1) include, moving the arms in opposition to the legs (skip criteria 2, 92% prevalence of failure), arms flex and swing forward to produce force (hop criteria 3, 95% prevalence of failure), failing to extend the arms forcefully (horizontal jump criteria 2, 77% prevalence of failure), failing to step forward with foot opposite throwing hand (underhand throw criteria 2, 84% prevalence of failure) and (overhand throw criteria 3, 90% prevalence of failure). Moreover, a large proportion of CwID struggled when skills required them to rotate their body (overhand throw criteria 2, 90% prevalence of failure) and (one hand strike criteria 1, 88% prevalence of failure). The findings presented in this study are consistent with those found in studies also assessing the behavioural components of skills however, these studies reported on TDC of various ages including (a) Irish adolescents, [19] (b) Australian pre-school children [28] and (c) British preschoolers and primary school children [25–27].

To summarise, these findings suggest that the areas in which CwID experience the most difficulty are the “timing and coordination of movement sequences” [38]. CwID have the ability to perform skills that are less complex and have reduced reliance on cognitive functioning [38], however when numerous body parts are required to move simultaneously more errors in FMS proficiency arise. The analysis of individual skills at the behavioural component level has highlighted some of the environmental and task constraints of FMS development that can be used by coaches/teachers to develop and tailor more specific, effective interventions within and across FMS that target these weaknesses [19,25,26]. This in depth understanding of FMS at the behavioural component level will provide a key focus for gatekeepers to direct them to the skill components requiring improvement, in addition to assisting them in modifying task achievements to have the greatest impact on FMS progression for CwID and help this population overcome the proficiency barrier [9,12,25]. To our knowledge, this is the only study of its kind to evaluate FMS development at a behavioural component level for CwID, so this area still remains relatively unexplored and should be investigated in future studies.

### Gender and age

Despite research describing balance as ‘the most basic skill’ of the FMS components [2,3], the overall balance scores for participants in this study are particularly poor compared to locomotor and ball skills scores (Table 2). This finding is in line with other studies analysing CwID [16,23,39]. In addition, there is a large and significant difference between the two groups in the subtest of balance where the girls achieved significantly higher mean scores (p < .001), particularly in the walking on the line skill where the effect size is 0.77. It is important to note that despite the girls outperforming the boys, overall as a cohort their balance proficiency levels are extremely poor and far behind what is expected. Improvements in static and dynamic balance for CwID is vital, not only to improve overall FMS proficiency but also to increase stability during activities of daily living and thus decrease risk of falls [40].

The literature demonstrates that in relation to the balance skills of school aged children, girls outperform boys, in particular with the single leg stand [41,42]. Many articles have demonstrated that gender differences in FMS performance can be accounted for by the activities that boys and girls participate in; these are often determined by social factors such as family, friends and also the physical environment [10,28]. In terms of the developmental timelines of girls and boys, it is important to note that children in this study are between the ages of 4–12 years and therefore, possess similar biological characteristics prior to reaching puberty including body composition, limb length, strength and genotype [43]. Furthermore, girls tend to participate more in activities such as gymnastics and dance which strengthen locomotor and balance skills, while boys often participate in sports and activities like football which heavily involve ball skills [10]. Ultimately, these studies refer to TDC, there is a clear lack of evidence focusing specifically on gender differences of FMS in CwID. Further research is required in order to confirm if the gender differences in FMS documented for TDC are the same for CwID.

When considering specifically CwID, previous studies on FMS proficiency often only focuses on the locomotor and ball skills subtests, rather than a holistic view of all three subtests. Results from a study by Rudd et al., [44] indicate that children’s balance skills will not improve by solely focusing on performance of locomotor and object control skills. Debates within the literature exist discussing whether balance is deemed as a FMS or whether it is simply postural adjustments to different environments [25]. In this paper, we hold the view that balance is a type of FMS, aligning with Newell’s [34] in-depth analysis of FMS development. This perspective offers a comprehensive understanding of FMS proficiency among CwID. It is evident from the balance results displayed in Table 2 that FMS interventions need to focus on balance skills for CwID, in addition to incorporating locomotor and ball skills training.

## Conclusions and implications for future research

The results of this study highlight the variation in mastery of skills at a behavioural component level for the first time amongst CwID. The weaknesses presented across the FMS may indicate that CwID are experiencing a ‘proficiency barrier’ that could hinder their involvement in lifelong sport and physical activity. It is important to highlight that all participants in this study take part in the Special Olympics Ireland Young Athletes Programme and therefore attend weekly sports training. Going forward, it would be ideal to have access to a control group of CwID to gain further indication of the role and impact of the Special Olympics Ireland Young Athletes Programme. Additionally, this would be vital to investigate any further deficiencies in FMS proficiency that may exist for CwID who do not participate in any sports or physical activity outside of school. It is hypothesised by Seefeldt [5] that children may need to meet a certain mastery level in FMS in order to progress and acquire more complex skills e.g. TMS and SSS [6]. Overcoming this proficiency barrier phenomenon is particularly important for CwID in order for them to gain the health enhancing benefits from lifelong physical activity participation and improve their overall quality of life. The findings indicate the importance of coaching/teaching each individual skill component and presenting CwID opportunities to repeat the movements multiple times in order to achieve skill mastery. Coaches and Teachers could adopt a constraints-led approach to teaching FMS in order to provide CwID the chance to succeed when practising FMS. Balance appears to be the weakest component of the three FMS subtests and future interventions need to account for this. Studies focusing on TDC have empirically tested the motor skill proficiency barrier, however this has not yet been done in CwID. Future studies could test the hypothetical barrier to investigate its impact on FMS development in CwID.

## Supporting information

S1 Fig Tetrachoric correlation matrix of the behavioural components of each skill. All coloured areas in the matrix are p < .01. (TIF)

S1 Dataset (XLSX)
