# Supplementary figures and images for: Exploring the motor skill proficiency barrier among children with intellectual disabilities: Analysis at a behavioural component level
Source: PLoS One. 2023 Nov 28;18(11):e0288413. doi: 10.1371/journal.pone.0288413 (PMC10683983; doi:10.1371/journal.pone.0288413)

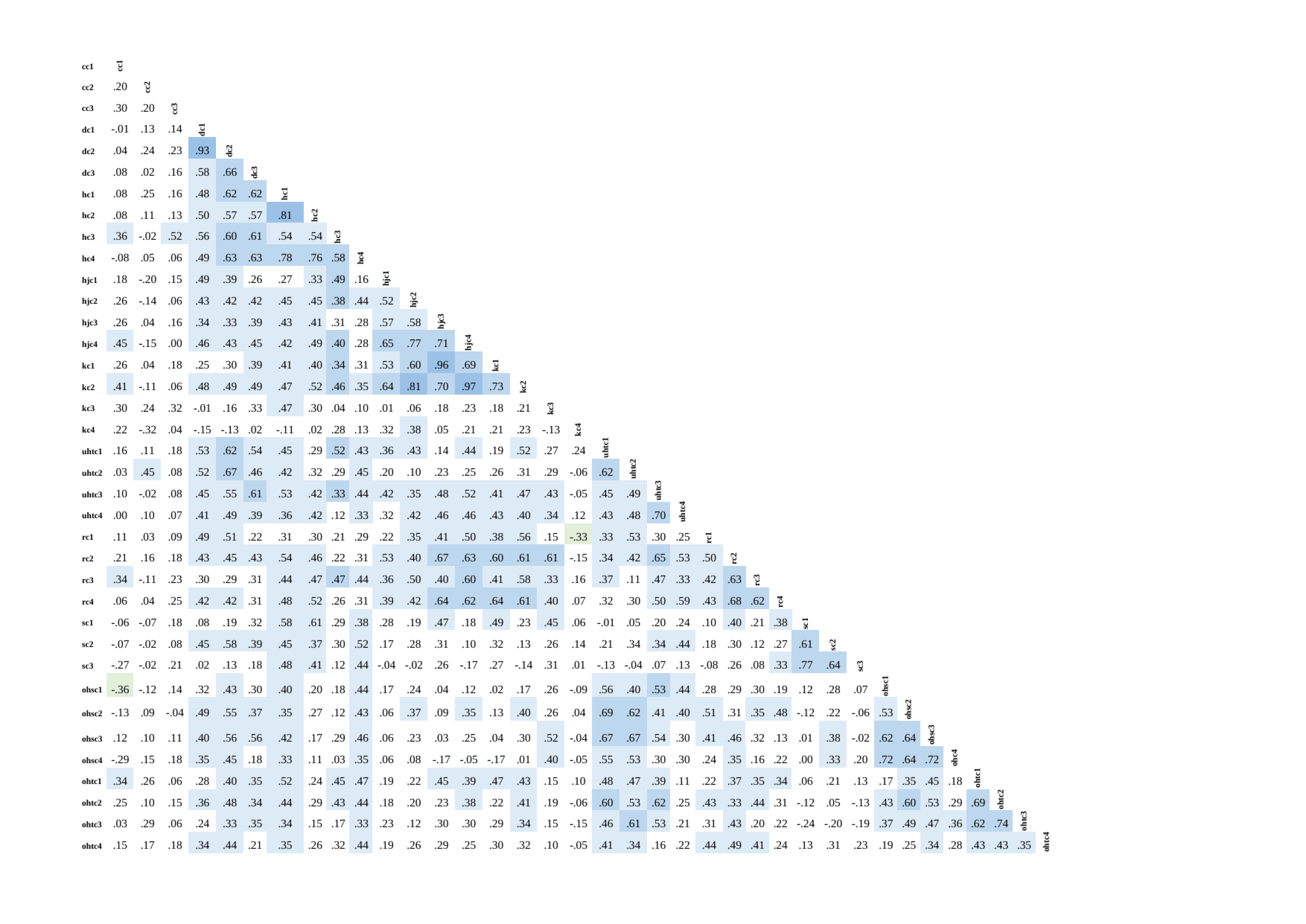

Supplement: S1 Fig — (TIF) [file pone.0288413.s001.tif]
